# Research progress of biomaterials and innovative technologies in urinary tissue engineering

**DOI:** 10.3389/fbioe.2023.1258666

**Published:** 2023-08-14

**Authors:** Liwei Duan, Zongliang Wang, Shuang Fan, Chen Wang, Yi Zhang

**Affiliations:** ^1^ The Second Hospital, Jilin University, Changchun, China; ^2^ Key Laboratory of Polymer Ecomaterials, Changchun Institute of Applied Chemistry, Chinese Academy of Sciences, Changchun, China

**Keywords:** biomaterials, technology, reconstruction, tissue engineering, urinary

## Abstract

Substantial interests have been attracted to multiple bioactive and biomimetic biomaterials in recent decades because of their ability in presenting a structural and functional reconstruction of urinary tissues. Some innovative technologies have also been surging in urinary tissue engineering and urological regeneration by providing insights into the physiological behavior of the urinary system. As such, the hierarchical structure and tissue function of the bladder, urethra, and ureter can be reproduced similarly to the native urinary tissues. This review aims to summarize recent advances in functional biomaterials and biomimetic technologies toward urological reconstruction. Various nanofirous biomaterials derived from decellularized natural tissues, synthetic biopolymers, and hybrid scaffolds were developed with desired microstructure, surface chemistry, and mechanical properties. Some growth factors, drugs, as well as inorganic nanomaterials were also utilized to enhance the biological activity and functionality of scaffolds. Notably, it is emphasized that advanced approaches, such as 3D (bio) printing and organoids, have also been developed to facilitate structural and functional regeneration of the urological system. So in this review, we discussed the fabrication strategies, physiochemical properties, and biofunctional modification of regenerative biomaterials and their potential clinical application of fast-evolving technologies. In addition, future prospective and commercial products are further proposed and discussed.

## 1 Introduction

For decades, the major challenges that urologists are eager to overcome include the injury and function loss of urinary tissue caused by congenital disorders, inflammation, tumor, trauma, and degenerative diseases (Drewa et al., 2012; Zhang et al., 2020). For a long time, as a gold standard for clinical treatment, autologous tissue has played an important role in replacing damaged urinary system tissues, such as bladder mucosa, oral mucosa, and genital skin (Jiang et al., 2018). However, its prominent limitations lie in the insufficient source of donor tissue, immune rejection, the essential difference of structure and function between the donor site and the transplantation site, as well as related various complications, including infection, stone formation, metabolic disorder, excessive mucus production, and so on (Wang et al., 2021a; Chen et al., 2021). To address these problems, as a result of the rapid development of regenerative medicine and tissue engineering, damaged urinary tissues and organs can now be replaced and/or regenerated. By means of biomaterials, cells, and growth factors, urologic tissue engineering technology aims to repair tissue defects, reconstruct new tissues, restore organ functions, and finally improve the life quality of patients (Chan et al., 2020).

There are two parts to the urinary system: the upper urinary tract and the lower urinary tract. The upper urinary tract consists of the kidney and ureter. The bladder and urethra make up the lower urinary tract (Liu et al., 2021). In the urinary system, urine is formed in kidney and then passes through ureter into bladder. Ureter is connected to bladder through the posterior lateral opening of bladder, while bladder is connected to urethra through its anterior opening. The bladder wall consists of four layers, including the urinary tract epithelial layer facing the bladder cavity, the connective tissue layer, the muscular layer, and the outer surface serosal layer (Sulob et al., 2021). The dysfunction or disorder of the normal urinary system requires reconstructive treatment to reduce urologic incontinence, preserve the storage function of the bladder, and minimize the risk of kidney injury (Shamout et al., 2023). Large or complex urinary injuries require surgical management and the choice of repair depends on the site of injury and timing of identification. Surgery treatment often include repairing urethral strictures or bladder damaged tissues, or repairing an abnormal bladder, and so on (Zelivianskaia et al., 2021; Fu et al., 2023). However, the repair of urinary bladder injury is often associated with some short- and long-term complications (Cohen et al., 2016). It is interesting to note that biomaterial scaffolds in tissue engineering have been presenting biomimetic substitutes for reconstructing the complex structure and function of the native urinary system. The ideal biomaterial scaffolds for the urinary system should first have appropriate mechanical strength and flexibility to resist the physiological pressure of continuous dynamic changes of urinary filling and emptying and facilitate cell migration and differentiation (Ajalloueian et al., 2018). Along with the ingrowth of newly formed tissue, the scaffolds would slowly degrade in a specific period. In addition, cells or growth factors can also be seeded or impregnated in the scaffolds to efficiently re-organize the native extracellular matrices physiologically (Adamowicz et al., 2019). Because of these aspects, kinds of acellular matrix, as well as natural and synthetic polymer scaffolds have been developed successively together with some advanced technologies towards urologic reconstruction.

**FIGURE 1:**
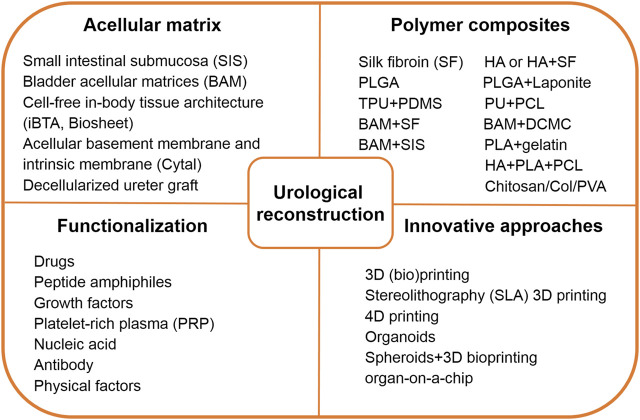
Brief summary of functional biomaterials and innovative technology toward urological reconstruction. Abbreviations: HA, hyaluronic acid; PLGA, poly (lactic-co-glycolic acid); TPU, thermoplastic polyurethane; PDMS, polydimethyl siloxane; PU, polydimethyl siloxane; PCL, poly (ε-caprolactone); BAM, bladder acellular matrix; SF, Silk fibroin; DCMC, dialdehyde carboxymethyl cellulose; SIS, small intestinal submucosa; PLA, poly (lactic acid); Col, collagen; PVA, polyvinyl alcohol.

This review aims to summarize the latest progress in functional biomaterials and fast-evolving technologies reported in the past 3 or 4 years for the repair and regeneration of the urinary system (Figure 1). The fabrication techniques and specific characteristics of a tissue-derived acellular matrix, natural and synthetic polymer scaffolds will be discussed first to address the urgent need of developing biomimetic materials and grafts for urinary reconstruction. Then, we try to pay special attention to the new strategy of biological modification of scaffolds using bioactive substances or inorganic components to improve the quality of reconstruction. Some innovative platform technologies for the regeneration of urologic tissues are also summarized. Finally, future prospects and clinical application potential of these biomaterials and techniques are given.

## 2 Biomaterials

Biomaterial-based urinary tissue engineering strategies are serving to address the growing demand for urological reconstruction (Sharma and Basu, 2022). Biomaterials and scaffolds should first stabilize structures with suitable mechanical properties, resist the physiological contraction and expansion pressure, and supporting cell migration (Lawkowska et al., 2022). Timely degradation is also necessary to promote the efficient ingrowth of new tissues (Lin et al., 2023). Regarding all these aspects, it includes acellular matrix, natural and synthetic polymer materials, and composite materials, etc.

### 2.1 Acellular matrix

Although acellular scaffolds have shown some limitations such as cytotoxic and inherent immune responses, many kinds of acellular matrices derived from various sources play a crucial role in urinary tissue regeneration by imitating natural extracellular matrix (ECM), including small intestinal submucosa (SIS), bladder acellular matrix (BAM), acellular corpus spongiosum (ACSM), and acellular amniotic membrane (Adamowicz et al., 2019). It is also common to use porcine SIS and BAM for urethra reconstruction in fundamental research and human clinical application (Ribeiro-Filho and Sievert, 2015; Cao et al., 2019). Chen and colleagues treated two patients with ureteral strictures using a semi-tubular 4-layer decellularized SIS matrix (Cook Co., Ltd. United States), and summarized its clinical effects (Xu et al., 2020a). They sutured the strip-shaped SIS matrix (4.0 cm long and 2.0 cm wide) onto the open ureter. Two months after surgery, ureteroscopic examination of the implanted SIS matrix showed that the internal surface of the implant was covered by mucosa, and the sutured lumen became narrow. After 4 months, the SIS matrix partially degraded, and the narrow ureteral stricture was covered by rough mucus. The SIS matrix was completely degraded after 6 months with rough mucosa covered on the ureteral stricture. Twelve months later, the SIS matrix was completely degraded, and no stricture was noted anymore with smooth and clean mucosal surfaces. No urinary leakage or infection was observed and the urography showed that ureteral anastomosis was no longer narrow. Serum creatinine levels returned to normal levels similar to those before surgery. The CT examinations of the urinary tract showed no signs of hydronephrosis. This SIS matrix is expected to serve as a substitute for ureteral reconstruction.

**FIGURE 2:**
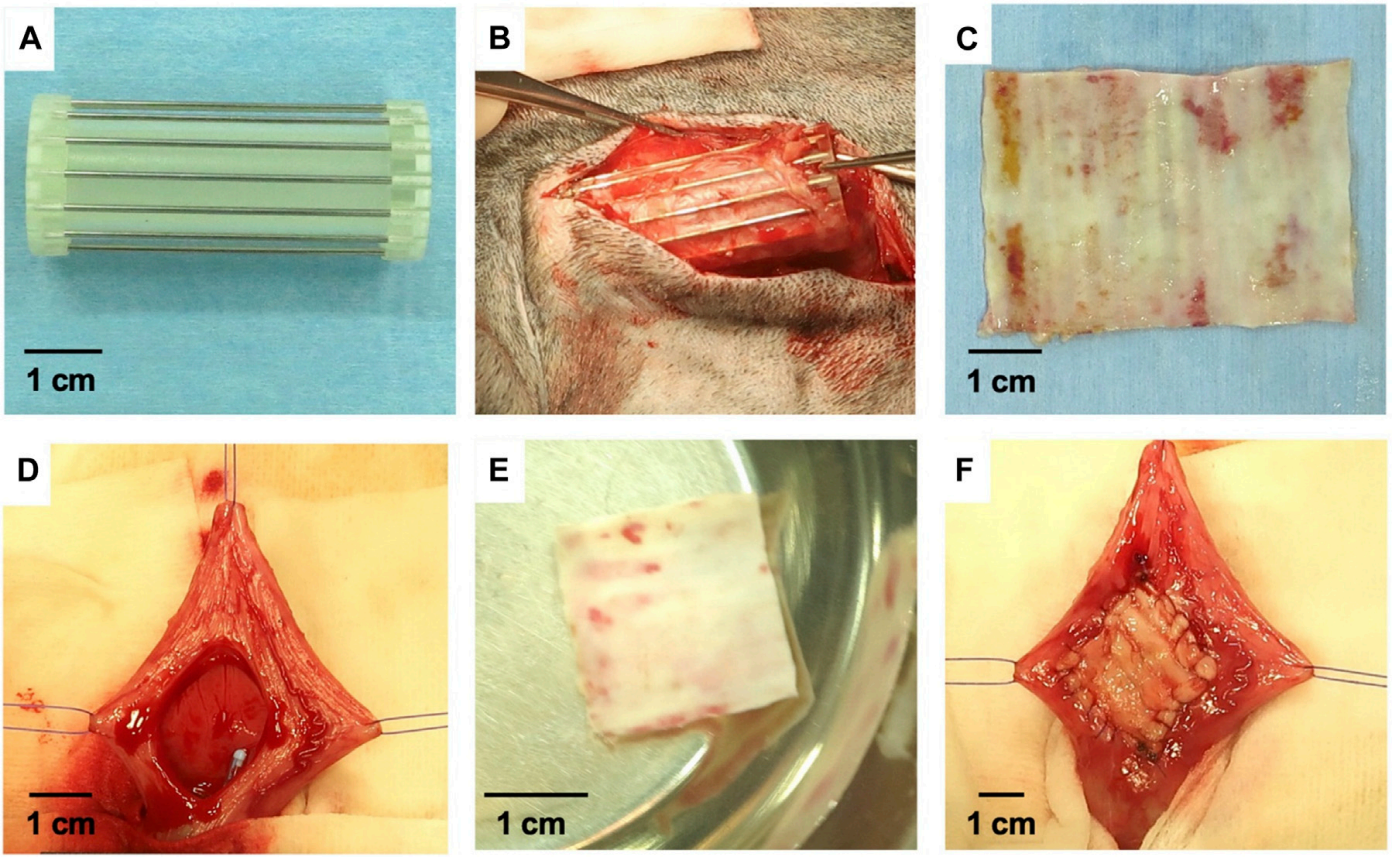
The preparation process and implantation of Biosheet implants. **(A)** Mold for the preparation of Biosheet. **(B)** Mold was completely encapsulated within the Biosheet tissues 8 weeks after implantation. **(C)** The fragile and redundant tissues covered on the Biosheet was clearly removed. **(D)** Urinary bladder wall after dissection (2 × 2 cm) **(E)** Trephined Biosheet (2 cm in diameter) **(F)** Urinary bladder after implantation of Biosheet. *Regenerative Therapy.* 2020; 15: 274–280. Copyright 2020, with permission from Elsevier (Iimori et al., 2020).

Recently, an emerging tissue engineering technology has been developed for the preparation of cell-free in-body tissue architecture (iBTA), which was used to produce autologous collagenous tissues with appropriate shape and mechanical properties via an alternative mold (Furukoshi et al., 2019; Makoto Komura et al., 2019; Terazawa et al., 2020). For this technique, decellularization is no longer required, thus avoiding the complex *in vitro* cell seeding processes in a clean laboratory environment. The tissue sheets obtained using the iBTA technique were named “Biosheet”, which was composed of fibroblasts and ECM rich in collagen type I (Col) (Takiyama et al., 2016). In one following study, Iimori et al. implanted porous cylindrical molds subcutaneously in dogs for 8 weeks to prepare Biosheet implants for bladder reconstruction (Figure 2) (Iimori et al., 2020). The molds consisted of several stainless steel slits that were inserted into an acrylic tube with caps. After 8 weeks, flexible rectangular Biosheet implants composed of collagen and fibroblasts with a length of 3 × 5 cm and a thickness of 1 mm were harvested by longitudinally cutting the tubular tissues (Suzuki et al., 2018). Then, a piece of full-thickness ventral wall of the urinary bladder with a size of 2 × 2 cm was removed and immediately sutured with the same-sized freshly prepared autologous Biosheet implants. After 4 and 12 weeks post-implantation, the Biosheet implants were extracted for histological examination. During the entire observation period, no urine leakage, stones, hematoma, calcification, or metaplasia were found on urography and ultrasonography examination. At 4 weeks, the Biosheet implants were covered by a multicellular layer of regenerated transitional epithelium and new blood vessels, forming partially substituted mucosa. There were visible connective tissue, neovascularization, infiltration of lymphoid, macrophage, and inflammatory cells. After 12 weeks, the boundary between the Biosheet implants and the natural bladder was indistinguishable, retaining a deep submucosa. Well-differentiated urothelial layers and submucosa organization could be seen on the Biosheet implants, which were better than at 4 weeks. Alpha-smooth muscle actin (α-SMA) positive stained spindle cells infiltrated into the boundary between the Biosheet implants and the natural muscle tissues. Few inflammatory cells, no necrosis or calcification were noted for the Biosheet implants. The results of this study indicated that the autologous Biosheet implants had good biocompatibility, and its epithelium remodeling as well as neovascularization were beneficial for bladder reconstruction in dog models, without any harmful signs of chronic inflammation or rejection. The Biosheet implants would be applicable as a candidate substitute in the clinical reconstruction of full thickness bladder wall.

In addition, Cytal is one of the commercial acellular sheet scaffolds extracted from the basement membrane and intrinsic membrane of the porcine urinary bladder by ACell Inc. (Gilbert et al., 2005). Its ingredients include growth factors, glycosaminoglycans, and collagens. Initially, Cytal was designed to treat wounds of partial thickness and full thickness, such as surgical and traumatic wounds, pressure ulcers, and diabetic ulcers (Valerio et al., 2015). To understand its application in urethral tissue reconstruction, Huen et al. (2022) summarized their preliminary experience in implanting Cytal in children who underwent ventral curvature correction during the repair of proximal hypospadias. They reviewed surgeries during 2020 and 2021 within 4 surgeon hypospadias databases in a single institution. Ten male patients who were implanted with single-layer Cytal grafts through ventral curvature correction all showed straight erections. In addition, from the perspective of cost, the price of Cytal graft is favorable to patients compared with other commercial products. And donor site morbidity would also not be caused by the implantation of Cytal grafts.

Additionally, Sabetkish et al. (2020) prepared decellularized bladder scaffolds and an inverted hourglass technique was used to assess the feasibility of scaffolds for double-sized bladder augmentation in rabbits. For decellularization, harvested urinary bladders were administered with 2% sodium dodecyl sulfate (SDS) for 6 h, soaked in Triton X-100 for 4 h, and washed in phosphate-buffered saline (PBS) for 2 h. The hourglass technique involved suturing the bottom of the acellular scaffold to the bottom of the natural bladder through the serosal layer to prevent bladder exposure. The control group underwent resection of the dome muscle and mucosa of the bladder, and the acellular scaffold was directly sutured to the bladder wall for bladder augmentation. After 1, 3, and 9 months, no shrinkage, infections, and reactive were observed for macroscopic view in the hourglass technique group compared with the natural bladder tissue. However, macroscopic observation of the control group showed significant shrinkage, rejection, and fall from the bladder wall. After 3 months of implantation, the implanted scaffolds promoted epithelium and muscle regeneration, with high levels of immunohistochemistry (IHC) staining, and no significant difference compared to 9 months after surgery. On the contrary, a higher grade of fibrosis was observed for the control group from the histopathological evaluation. In all cases, the implantation of fully decellularized bladder scaffolds resulted in a successful double-sized bladder autoaugmentation for patients with a small bladder capacity. In comparison with synthetic materials and other natural scaffolds, this material has proven to be a significant alternative material.

Similarly, Moreno-Manzano et al. (2020) seeded human adipose-derived stem cells (ADSCs) on decellularized rat bladder matrices and then implanted them into rats with partial cystectomy to regenerate the bladders. The authors found that the implantation of the ADSC-contained bladder matrices significantly promoted the recovery of the urothelium, the organization of smooth muscle layers, the creation of new blood vessels and nerve innervation. The paracrine effect of ADSCs on the decellularized bladder matrices made the recellularized matrices to be a potentially effective bladder substitute. A similar strategy has also been performed by Vishwakarma et al. (2020), and the authors fabricated cell-laden decellularized goat bladder scaffolds for bladder reconstruction. Subsequently, human umbilical cord blood-derived mesenchymal stem cells (hUCBMSCs) were seeded to prepare bioactive decellularized bladder scaffolds for bladder augmentation. The composition, architecture and mechanical characteristics of the decellularized bladder scaffolds were well retained. The adhesion and proliferation of the seeded hUCBMSCs on the scaffolds were significantly improved at 14 days. So the prepared decellularized goat bladder scaffolds represented a simple and controlled fabrication process of biological tissue-specific substitutes and could provide a biocompatible microenvironment for transplanted MSCs in bladder reconstruction.

Correspondingly, decellularized ureter graft has also been developed by Sabetkish et al. (2022) for rat bladder augmentation. In this study, the ureteral samples were donated by four adult male patients who volunteered to donate their kidneys. The ureters were decellularized by successively using 2% SDS and 2% Triton X-100, PBS washed afterwards. The microstructure of the acellular extracellular matrix composed of well-organized type I and III collagen fibers was well maintained, with a tensile strength of 5.1 × 10^3^ kN, similar to that of normal ureter (5.5 × 10^3^ kN) and bladder tissue (5.8 × 10^3^ kN), and no nucleus was retained. No contractions, infections, and stone formation were observed in the animals implanted with the decellularized ureters. It was worth noting that all the groups did not experience fibrosis or degeneration. After 9 months, complete regeneration of the bladder wall was achieved by implanting the decellularized ureters, and the boundary between the host bladder and the decellularized ureter was no longer easily distinguished. The decellularized urethral grafts in this study have broad potential as biocompatible scaffolds for cell ingrowth and morphological formation of bladder tissues (Singh et al., 2018; Haeublein et al., 2022).

Undoubtedly, acellular tissue matrices have unique advantages as they possess the inherent components of natural ECM to support cell growth and differentiation. Natural proteins and growth factors, including collagen, vascular endothelial growth factor (VEGF), and fibroblast growth factor (FGF), etc., can be retained in the matrix. The problems they need to overcome are cytotoxic, fibrosis, infection, and immune response (Hughes et al., 2016; Wang et al., 2023a).

### 2.2 Natural and synthetic materials

The outstanding advantage of natural and synthetic materials is that they are easy to manipulate and process to achieve the required functions (Wang et al., 2023b; Sung et al., 2023). Many of them have been approved for commercial applications, including poly(glycolic acid) (PGA), poly(lactic acid) (PLA), and poly(lactic-co-glycolic acid) (PLGA), etc., (Alvarez-Mendez et al., 2023). The significant drawback lies in inducing foreign body reactions to the host and insufficient angiogenesis (Zhang et al., 2022).

**FIGURE 3:**
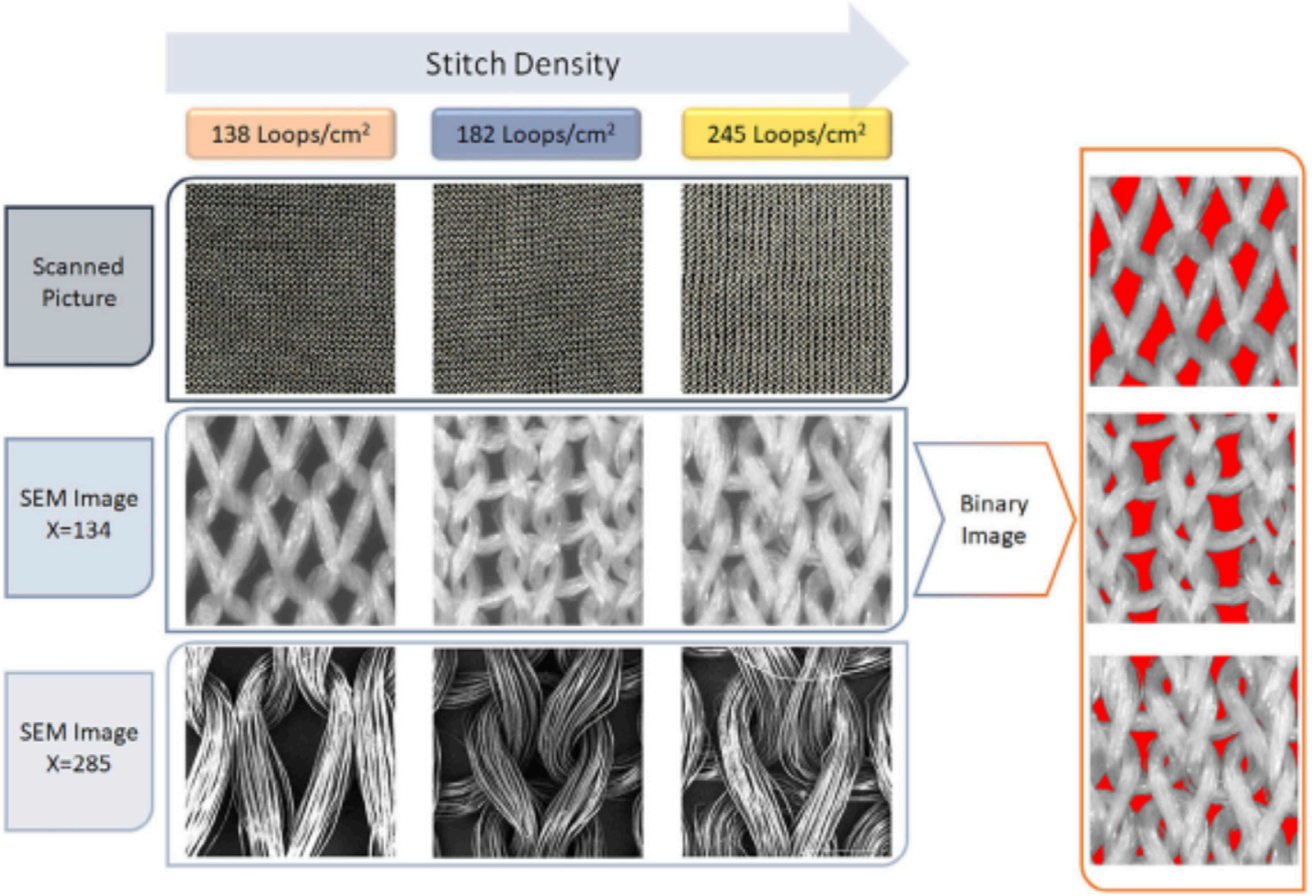
Scanning electron microscope (SEM) images of the silk fibroin weft-knitted scaffolds, and binary images of silk fibroin weft-knitted. *Polymers Advanced Technologies.* 2021; 32: 2367–2377. Copyright 2021, with permission from Wiley (Khademolqorani et al., 2021).

Among them, silk is a natural protein fiber, and its superior mechanical properties, biocompatibility, as well as degradability make it very suitable for bladder reconstruction (Bhattacharjee et al., 2017; Nguyen et al., 2019; Li and Sun, 2022). Tavanai and others produced weft-knitted silk fibroin (SF) scaffolds (138, 182, and 245 loops/cm^2^ of stitch density respectively), and evaluated their suitability for bladder regeneration (Figure 3) (Khademolqorani et al., 2021). Weft-knitted scaffolds have the following structure characteristics: perpendicular connecting wale and course loops (Eltahan et al., 2016; Cai et al., 2020; Meng et al., 2020). The authors knitted scaffolds with varying stitch densities using a knitting machine that can adjust tension. By increasing the stitch density, the porosity and pore size of the scaffolds decreased. The weft-knitted scaffolds showed an average tensile strength of 7–7.9 MPa and 8.1–8.8 MPa in the course and wale directions, respectively. After co-culturing with NIH-3T3 fibroblasts for 1, 2, 4 and 6 weeks, the tensile strength and ultimate strain of the weft-knitted scaffolds were higher than those of pure scaffolds at all time points. In addition, among all the three groups, the SF scaffolds with 182 loops/cm^2^ stitch density exhibited the most similar stress-strain behavior to the natural porcine bladder, which meant it could simulate the process of bladder filling and urination. Therefore, its excellent performance makes it a promising candidate for bladder reconstruction.

**FIGURE 4:**
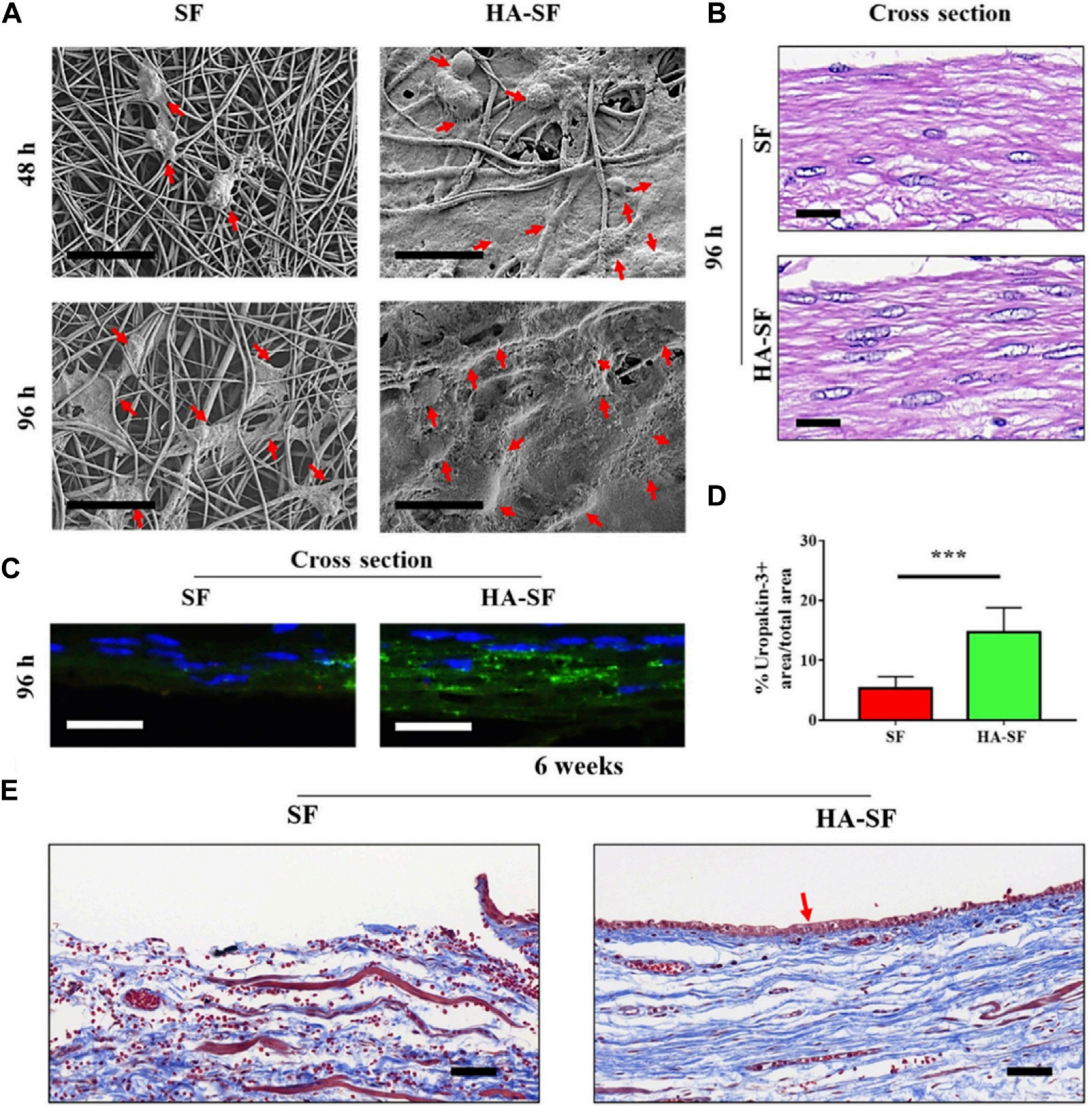
Cell behavior on silk fibroin (SF) and hyaluronic acid (HA)-SF nanofiber films. **(A)** SEM images of primary urothelial cells (UCs) on the inner wall surface of SF and HA-SF nanofiber after 48 and 96 h post-seeding. Red arrows indicate well-spread UCs. **(B)** Hematoxylin and eosin (HE) staining of the cross section of cellularized SF and HA-SF nanofiber films. **(C)** Fluorescence staining of the cross section of cellularized SF and HA-SF nanofiber thin films using uropakin-3 (green) and nuclei (blue). Scale bars: 20 μm **(A–C)**. **(D)** Statistical data of uropakin-3 positive expression of primary urethral UCs seeded on different nanofibers (three random fields per sample, n = 4 samples per group). ***p < 0.01. **(E)** Masson’s trichrome staining of the cross section of the tubular SF and HA-SF nanofibers implanted for 6 weeks. Collagen (blue), smooth muscle, and erythrocytes (red). Red arrow indicates that the captured host endogenous urothelial cell (UC) is evenly distributed in the lumen of HA-SF scaffold. Scale bars, 20 μm. *Bioengineering and Translational Medicine.* 2022; 7: e10268. Copyright 2021, with permission from Wiley (Niu et al., 2022).

Hyaluronic acid (HA) has been reported to have the ability to bind to cell surface receptors and recruit urethral stem cells to stimulate cell behavior and regulate tissue formation (Aruffo et al., 1990; Niu et al., 2021). In one study, a biomimetic tubular nanofiber scaffold with appropriate cell-binding motifs and sufficient mechanical properties was electrospun by integrating HA with natural SF protein to promote urethra epithelialization (Figure 4) (Niu et al., 2022). To further modulate the topological structure of SF and HA-SF nanofiber scaffolds, chemical crosslinking was carried out by immersing in ethanol and 1-ethyl-3-[3-dimethylaminopropyl] carbodiimide hydrochloride (EDC) solution. SF nanofibers showed a tightly interlinked nano-topography, while HA/SF nanofibers exhibited interconnected network decorating gel-like surface morphology, similar to the morphology of natural urethral epithelium. When urothelial cells (UCs) were cultured on the nanofibers, the cells preferentially adhered to the surface of HA/SF, with elongated morphology, uniform distribution, and ingrowth, which was confirmed by scanning electron microscopy (SEM) and cross-section observation of hematoxylin and eosin (HE) staining. The immunofluorescence staining for the proliferation marker, Ki67, and cell counting kit-8 (CCK-8) results showed that UCs proliferated well on the HA/SF nanofibers. The higher density positive green fluorescence of uropakin-3 on the HA/SF nanofibers confirmed its waterproof ability and anti-injury function for urothelial barrier restoration. Eight weeks after implantation in the rabbit transected urethral defect, the HA/SF group exhibited a slight lower urine flow rate (8.4 ± 0.2 mL/s) than that of before implantation (8.9 ± 0.2 mL/s), while the SF group showed the lowest level (6.6 ± 0.2 mL/s). By observing the Masson’s trichrome staining of the regenerated urethral tissue, a dense UC layer and new collagen deposition were formed along the HA/SF scaffold. After 14 weeks, stratified epithelial remodeling occurred in the regenerated urothelium of the SF and HA/SF groups. While the thickness of the regenerated middle and surface layer in the HA/SF group (43 ± 3 μm) was similar to the normal urothelial epithelium and thicker than that of the SF group (26 ± 1 μm). The upregulation of double fluorescent staining for K5 and uroplakin-3 further confirmed that HA/SF nanofibers promoted the regeneration of K5 positive cubic cell layer in the urinary tract epithelium basal and outermost thin layer of uroplakin-3 positive flat cell, indicating that the effective remodeling of the blood urine barrier was similar to that of the healthy urothelial barrier. In addition, in the HA/SF nanofiber group, smooth muscle tissue remodeling and angiogenesis confirmed that lumen and myoepithelial cells were successfully recruited from adjacent areas, thus producing a higher proportion of smooth muscle bundles and α-SMA immunofluorescence staining positive areas, as well as higher levels of CD31 positivity in the regenerated urethra. This biomimetic scaffold provides a synergistic effect of nano-topography and biophysical cues, and promotes efficient endogenous regeneration by presenting meaningful aspects different from SF protein scaffolds.

As an alternative, PLGA-based nanofiber scaffold was electrospun to regulate the differentiation and regeneration of smooth muscle cells (SMCs) (Mirzaei et al., 2019a). After inducing and culturing the iPSCs on the PLGA scaffold for 2 and 3 weeks, genes related to SMCs were upregulated, such as myosin heavy chain (MHC), smooth muscle 22 alpha (SM-22α), Calponin-1, α-SMA, and Caldesmon1. This indicated that induced co-culturing of human iPSCs on the PLGA nanofibrous structure with an appropriate elasticity and strength, simulating the structure and function of natural bladder extracellular matrix, had great differentiation potential toward SMC, effectively promoting SMC differentiation and cellular penetration, and enhancing bladder tissue regeneration.

Similarly, Salem et al. prepared a tissue-engineered construct by seeding SMCs onto a commercial three-layer PLGA woven mesh (VICRYL, Ethicon Inc., United States) and then suturing it to rat bladder (Salem et al., 2020). After 90 days of surgery, radiography showed that the bladder reconstructed with the tissue-engineered construct presented a normal oval appearance and a smooth and regular outline. The newly reconstructed bladder was generally circular and full in shape, with a size and shape similar to a normal bladder. The PLGA mesh was completely degraded and no longer visible. Histological observation revealed complete regeneration of the bladder wall, consisting of layered urothelium, submucosal blood vessels, and muscle bundles. The results of this study demonstrated that the implanted materials and delivered cells contributed to the newly formed muscle tissues and the ingrowth of urethral epithelium, thereby fully restoring the volume and function of the regenerated bladder.

Different from polymers, as a two-dimensional (2D) smectite clay with high aspect ratio, Laponite is a biocompatible and safe nanomaterial, which is easy to degrade in physiological environment (Tomás et al., 2018). In a study, Laponite nanoparticles were electrospun into PLGA nanofiber scaffolds to replicate the porosity and microstructure of the natural urethra tissue microenvironment (Wang et al., 2020). The higher content of Laponite in the composite scaffolds, the smaller fiber diameter, which might be conducive to cell adhesion and migration. The spontaneous fluorescence of Laponite particles in the composite scaffolds indicated their uniform dispersion within the scaffolds. The mechanical characteristics of the scaffolds and the degradation of the PLGA matrix increased with the increase of Laponite content. Human umbilical vein endothelial cells (HUVECs) could attached, spread, and grew well on the surface of the scaffolds. After 3 days of co-culturing with 1% Laponite/PLGA composite urethral scaffolds, the cell proliferation rate exceeded 100%, indicating that the structural characteristics of the composite scaffolds were similar to the natural extracellular, which is very useful for cell adhesion and proliferation.

Polyurethane and silicone rubber have also been evaluated for their biological safety and low toxicity in urinary reconstruction (Chew and Denstedt, 2004; Cosentino et al., 2012). To obtain better structural integrity and antibacterial properties, thermoplastic polyurethanes (TPU) modified by polydimethyl siloxane (PDMS) was prepared by dynamic crosslinking method as thermoplastic vulcanizate for resistance to deformation and processing in clinical treatment (Sharma et al., 2021). Compared with the physical or van der Waal forces assisted melt mixing process, the interface adhesion between PDMS and molten TPU matrix became stronger due to the *in situ* three-dimensional (3D) cross-linking (Damrongsakkul et al., 2003; Drupitha and Nando, 2017). The interlinks formed in the silicone phase endowed the composite with rigidity. For example, the ultimate tensile strength and elastic modulus of the dynamically crosslinked 80/20 TPU/PDMS composite (DT8P2) increased up to 15.7 ± 0.5 MPa and 8.4 ± 0.1 MPa compared with that of T8P2 without crosslinking (10.9 ± 2.0 MPa and 8.0 ± 0.2 MPa). The suture retention force (SRF) of T8P_VP_2, DT_bPEI_8P2, and DT_PAP_8P2 samples was 9.2 N, 14.1 N, and 13.8 N, respectively, which were far greater than the clinically acceptable minimum SRF value of 2 N to meet the reliable mechanical integration with the natural tissues (Billiar et al., 2001). Subsequently, a contact-killing antibacterial surface was generated by covalent modification of quaternized compounds, which included branched polyethylenimine (bPEI), 4-vinyl pyridine (4-VP), and bPEI-grafted-(acrylic acid-co-vinylbenzyltriphenyl phosphonium chloride) (PAP), on the TPU/PDMS composites. Compared with the TPU group, the bactericidal efficiency of T8P_VP_2 against *Escherichia coli* (*E. coli*), Methicillin-resistant *S. Aureus* (MRSA) and *Proteus mirabilis* (*P. mirabilis*) was 98.00%, 98.90%, and 99.37%, respectively, after 24 h of co-culture. The DT_bPEI_8P2 samples exhibited corresponding bactericidal efficiency of 99.50%, 99.90%, and 99.90%. While the DT_PAP_8P2 group showed 99.68%, 99.90%, and 99.84% of bactericidal efficiency, respectively. *E. coli* and *P. mirabilis* cultured on the T8P_VP_2, DT_bPEI_8P2, and DT_PAP_8P2 composites showed distorted morphology and permanent damage characteristics. The integrity of the MRSA cell membrane was disrupted, leading to leakage of cytoplasmic contents. The superior surface energy and wettability of the T8P_VP_2, DT_bPEI_8P2, and DT_PAP_8P2 samples contributed to the cell proliferation and characteristic morphology of L929 cells without inducing cytotoxicity. Therefore, the high-density cationic motifs functionalized surface of TPU/PDMS composites could inhibit the survival ability of bacteria in urinary tract infection and maintain appropriate *in vitro* cell behavior, serving as a potential biomaterial for developing medical urinary substitutes.

Broadly speaking, insufficient or slow vascularization of tissue-engineered urethral grafts often leads to complications such as urethral stricture and urethral obstruction, which hinder the success rate of urethral reconstruction in animal and clinical studies (Rashidbenam et al., 2019). It has been reported that the appropriate weakly hydrophilic polyurethane scaffolds could not only improve the cell compatibility and histocompatibility, but also rapidly realize the tissue vascularization of the scaffolds (Niu et al., 2014; Niu et al., 2017). In one study, thanks to the rapid vascularization of biodegradable urethral scaffold made of linear amphiphilic block-copolymer of polyurethane (PU-ran), Niu et al. successfully achieved functional regeneration of the Beagle urethra (Niu et al., 2020). The structure backbone of PU-ran copolymer was solution polymerized from hydrophilic poly (ethylene glycol) (PEG) and hydrophobic poly(ε-caprolactone) (PCL) segments in a controlled manner. As the content of PEG in the backbone increased, the semi-crystalline of PU-ran copolymers changed to an amorphous state, and the tuning chain length of PEG promoted elasticity and mechanical characteristics. The coordinated segments of flexible PEG and rigid PCL prevented crystallization, thus promoting the flexible and stretchable copolymer of PU-ran. The electrospun nanofibers composed of PEG (0.4 kDa) and PCL-diol (2.8 kDa) segments, abbreviated as E10-ran-C20, were softer and smoother than pure PCL. The seeded primary bladder epithelial cells (ECs) and primary SMCs could quickly adhere, migrate, and grow on the PU-ran copolymer nanofiber scaffolds. The E10-ran-C20 nanofiber scaffolds were advantageous to the adhesion and proliferation of urothelial tissue-derived cells, and the construction of 3D morphology (Sharma and Cheng, 2015; Cheng et al., 2019). The authors later found that the E10-ran-C20 nanofibers could induce higher expression of the epithelial cytokeratin (AE1/AE3+) in ECs, α-SMA+ in SMCs, and secretion of elastin. Subsequently, the E10-ran-C20 layered tubular matrix was used to deliver cells for mimicking the natural urethra in space and dimension, and the thickness of the epithelial ECM in the tube was approximately 500 nm, indicating good cytocompatibility. The engineered E10-ran-C20 scaffolds were implanted into a partial urethral defectin Beagle puppies to simulate common urethral injuries in young male children with traffic accidents and medical injuries. After 60 days *in vivo*, the E10-ran-C20 scaffolds gradually degraded and absorbed, ECs and SMCs delivered on the scaffolds continued to proliferate and secrete ECM (Orabi et al., 2013; Kullmann et al., 2017; Wang et al., 2017), and finally formed tubular lumen epithelium in the nanofiber scaffolds. The formation of smooth muscle tissues was due to the biocompatibility of SMCs with surrounding tissues and its growth in the biodegradable scaffolds. After 3 months, the mean urinary flow rates (AFR) and the average urethral diameter (MUD) of the E10-ran-C20 scaffolds were similar to those of the autologous grafts, allowing the contrast agent to pass through the entire reconstructed lumen. Masson’s trichrome staining showed that the newly formed epithelial cells in the urethral wall had a stratified epithelial morphology and were covered by smooth muscle tissue. A large amount of capillaries and small blood vessels were formed under the new epithelial basal. Collagen staining in the new urethra was more visible than that of autografts. The quantitate protein expression of smooth muscle (α-SMA+), urothelium (AE1/AE3+), and cell adhesion molecule (CD31) of the cell-seeded E10-ran-C20 scaffold implied that the regeneration of urethra was similar to that of the autograft group. The expression of cytokines and chemokines was elevated at the juction of the engineered E10-ran-C20 scaffolds, which facilitated the recruitment of host inflammatory cells, the neo-vessel formation, and the generation of regenerative microenvironment, contributing to the functional recovery of Beagles. This will help overcome potential difficulties, such as insufficient neovascularization within the grafts and associated urethral strictures, making it have great potential in treating many human urethral diseases (Lv et al., 2018; Sánchez et al., 2022).

### 2.3 Composite materials

To give full play to the high strength and elasticity of SF protein polymers and the preserved cellular components retained by BAM, Xiao et al. constructed a composite scaffold by combining double-layer SF film and sponge with BAM hydrogel and encapsulated ADSCs in the hydrogel for rat bladder augmentation (Xiao et al., 2021b). The composite scaffold was constructed by dropping the ASCs-encapsulated BAM hydrogel on the SF surface and continuing to incubate for 7 days. The biological components preserved in the bladder ECM provided a fluid and viscosity microenvironment for the uniform distribution of ASCs in the SF pores of the composite scaffold. It was worth noting that previously wrapping the constructed scaffold in the omentum for 7 days before anastomosing to repair the bladder defect achieved efficient vascularization and promoted the structural regeneration of the bladder wall. At 12 weeks after implantation, the SF/BAM/ASCs group formed a continuous urothelium layer and a large amount of smooth muscles. In addition, the ACSs delivered in the matrix hydrogel significantly regenerated smooth muscles, neurons, and blood vessels, thus restoring the function of rat bladder, which was proved by the immunofluorescence observation. The loaded ASCs also accelerated the degradation of the SF sponge in the composite scaffold.

The rapid degradation of BAM and the cytotoxicity of glutaraldehyde crosslinking reagent attracted Peng et al. (2019) attention, they used dialdehyde carboxymethyl cellulose (DCMC) with an appropriate molecular weight to crosslink BAM materials for bladder tissue engineering. The fixation of BAM by 30 mg mL^-1^ of DCMC obtained a cross-linked D-BAM composite with lower cytotoxicity, better mechanical properties, and resistance to enzyme degradation, while retaining the microstructure and biological components, including transforming growth factor beta (TGF-β), human keratinocyte growth factor (KGF), and glycosaminoglycans (GAGs). The bladder transitional epithelial cells cultured on the D-BAM composite were stimulated to secrete epidermal growth factor (EGF) and platelet-derived growth factor (PDGF), which was conducive to re-epithelialization. The D-BAM composite could inhibit the deposition of minerals in BAM tissues and possessed a prominent anti-calcification ability.

In another study, to overcome the poor expansibility and rapid degradation of decellularized extracellular matrixes (dECMs), including SIS and BAM, the transient crosslinking between dECMs and long-chain aliphatic molecules was developed. And dECMs were covalently linked to one end of the polymeric chains, while other molecules could interact at the other end through weak interactions, such as dipole-dipole forces (Sharma et al., 2022). Additionally, some other composite nanofibrous patches or scaffolds composed of PLA/gelatin or HA/PLA/PCL have also been generated to evaluate the critical potential of replacing damaged or diseased bladder and urethra (Liu et al., 2020a; Wang et al., 2022).

Among so many materials, acellular matrix can preserve the natural structure and composition of natural urinary tissues, while the natural or synthetic polymers or composite materials help to withstand mechanical loads during tissue regeneration process, even when the bladder is filled with urine (Table 1) (Hanczar et al., 2021). More importantly, they are also suitable for surgical procedures by providing structural rigidity, thereby resisting very high urinary pressure conditions (Zhu et al., 2022). The flexibility and stretchability of acellular matrix and artificial scaffolds are also crucial for adapting to increasing urine volume and stress (Chowdhury et al., 2021). Last but not least, suture resistance and impermeability are necessary for temporary storage of urine to avoid urine leakage (Pal, 1998; Ajalloueian et al., 2018; Lee et al., 2021). It can be expected that the promotion of the advantages and overcoming the disadvantages of different types of scaffold will make urinary tissue engineering better.

**TABLE 1:** The physical and mechanical properties of the biomaterials for uribary tissue engineering.

Biomaterials	Porosity (%) or diameter (nm)	Mechanical properties	Refs.
Decellularized bladders	None	∼88 KPa (Tensile strength)	Vishwakarma et al. (2020)
Decellularized ureters	None	(5.1 ×10^3^) kilonewtons (Young’s modulus)	Sabetkish et al. (2022)
Silk plain-weft knitted scaffolds	(89.9 ± 1.1) %	(7.1 ± 0.6) MPa in course direction and (8.1 ± 1.9) MPa in wale direction (Tensile strength)	Khademolqorani et al. (2021)
Hyaluronic acid and silk fibroin (HA-SF) nanofibers	(254 ± 13) nm	(0.83 ± 0.4) MPa (Young’s modulus)	Niu et al. (2022)
Poly(lactide-co-glycolide) nanofibers	(600 ± 400) nm	(11.32 ± 2.02) MPa (Tensile strength)	Mirzaei et al. (2019a)
Dynamically crosslinked thermoplastic polyurethanes modified by polydimethyl siloxane (TPU/PDMS)	None	(15.7 ± 0.5) MPa (Ultimate tensile strength)	Sharma et al. (2021)
Bi-layer silk fibroin skeleton	(62.67 ± 3.40)%, (81.98 ± 2.10)μm	None	Xiao et al. (2021b)

## 3 Biological functionalization strategies

### 3.1 Drugs or bioactive molecules

Natural biomolecules have been utilized for functionalization of transplant materials to overcome the limitations of contracture, stone formation, and smooth muscle regeneration (Mokhames et al., 2020; Zhang et al., 2021).

As a biotic component, curcumin has been selected to functionalize bladder scaffolds for protecting organs, inhibiting protease, and eliminating free radicals due to its anti-inflammatory, antioxidant and other biological activities (Bengmark, 2006; Anand et al., 2007). For example, Mokhames et al. (2020) prepared chitosan, collagen, and polyvinyl alcohol (chitosan/Col/PVA) nanofiber scaffolds doped with curcumin (nanofibers/curcumin), which had a randomly oriented and interconnected porous structure. In this composite scaffold, chitosan and PVA provided structural support, while Col served as a natural matrix that mimicked natural bladder tissue. In addition to the initial burst release of about 20% on the first day, the cumulative release of curcumin in 21 days increased slowly, and the release of curcumin from the nanofibers was up to 90%. The curcumin-loaded nanofibers exhibited the highest level of protein adsorption, cell attachment, and proliferation. Compared with the nanofibers and tissue culture plate groups, the nanofibers/curcumin group upregulated the expression of genes related to smooth muscle cells, including Calponin1, Caldesmon1, SM-22α, and α-SMA. Furthermore, immunocytochemistry staining analysis showed that the curcumin-incorporated nanofibers promoted the expression of α-SMA protein. Therefore, the natural bioactive substance curcumin in this study improved the differentiation of stem cells into bladder SMCs.

Procyanidin (PC) is a natural polyphenol and it was used to crosslink collagen materials for enhancing mechanical stability by forming hydrogen bonds with amide groups and also inhibit calcification (Han et al., 2003; Liu et al., 2013; Zhai et al., 2014; Balalaie et al., 2018). PC can also resist inflammation and oxidation, so it has been used in tissue engineering and the development of bioprosthetic heart valves (Schmidt and Baier, 2000; Zhai et al., 2006; Wang et al., 2015a; Wang et al., 2015b). To reduce contracture, enhance smooth muscle regeneration, and avoid stone formation of small intestine submucosa (SIS) materials during bladder regeneration, Zhang et al. (2021) prepared a PC-functionalized SIS scaffold (PC-SIS) with PC as a crosslinking agent. After crosslinking with a proper amount of PC, the diameter of collagen fibers in PC-SIS slightly increased, showing a deep brownish red color and a porous surface structure. Due to the cross-linking of PC, the PC-SIS exhibited excellent mechanical properties, good biocompatibility, slower degradation, and less formation of mineralized nodules. For the behavior of SMCs, PC-SIS supported cell growth and spreading, promoted cell organization, bundle-like structure formation, filopodia-like connection with adjacent cells, extracellular matrix deposition, and the SMC-related gene expression. When applied to full-thickness bladder defects, histological examination showed that the bladder tissue generated by PC-SIS contained more smooth muscle bundles than the SIS groups. The urodynamics analysis revealed that the PC-SIS materials had the same peak pressure, bladder volume, elastic modulus, and maximum load as the natural bladder tissue. The phenolic hydroxyl structure of catechin-containing PC with intermolecular hydrogen bonding ability provided a meaningful choice for cross-linking of SIS as a patch in bladder repair and reconstruction.

Self-assembled peptide amphiphiles (PAs) were synthesized to regulate the inflammatory microenvironment of urinary tract injury sites (Bury et al., 2014; Chan et al., 2021). By combining the hydrophobic collapse of the alkyl domains and the hydrogen bond of the β-sheet domains, the bioactive peptide epitopes of PAs could be specifically formed on the assembled nanofibers, which could be recognized by cell receptors or bound to other biological molecules to enhance function, such as anti-inflammatory and tissue regeneration (Hartgerink et al., 2001; Sohn et al., 2003; Silva et al., 2004; Webber et al., 2011). In one study, Hartgerink et al. (2001) first synthesized anti-inflammatory PAs (AIF-PAs) using solid peptide synthesis methods. Then SIS scaffolds were separately dip-coated in the AIF-PAs and then transplanted into the cystectomized bladder defects (Ashley et al., 2010). Compared with the unmodified group or the group modified with control peptides of AIF-PA6, the SIS scaffolds modified with the anti-inflammatory AIF-PA1 peptides significantly reduced the level of myeloperoxidase positive (MPO^+^) neutrophils, M1 proinflammatory macrophages (CD86^+^), proinflammatory cytokines of IL-1β and TNFα, while increased the expression of M2 regenerative/anti-inflammatory macrophages (CD206^+^) at the anastomotic site and regenerated wound region. The AIF-PA1-SIS scaffolds also facilitated higher levels of vascularization and collagen deposition, which meant faster progress of angiogenesis and urethral remodeling, and promoted the healing process of urethral defects. The same team also observed a similar regenerative effect of AIF-PA1 on bladder tissue regeneration. They found that the anti-inflammatory PA alternated the immune response of rat bladder augmentation, decreased the CD68^+^ macrophage and MPO^+^ neutrophil level, reduced the TNFα, IL-1β and M1 macrophage level, but increased angiogenesis (Bury et al., 2014). Therefore, the easy use of AIF-PAs nanofibers and their ability to regulate the inflammatory microenvironment have broad prospects in urethral reconstruction.

Bacterial infection and biofilm formation of implant materials are considered one of the main issues hindering tissue repair quality and affecting global health, and are receiving increasing attention (Sun et al., 2019; Gao W et al., 2021). Chrysophanol (CP) is a unique natural anthraquinone with broad-spectrum therapeutic effects. It was reported that CP of appropriate concentration could inhibit the formation of biofilm through bacterial quorum sensing (QS) (Ding et al., 2011; Prateeksha et al., 2019). Therefore, Singh and his colleagues utilized CP to biofunctionalize silver nanoparticles (AgNPs) to regulate the interaction between QS signals and AgNPs, reduce bacterial pathogenicity, and minimize the dosage of AgNPs that inhibited bacterial adhesion and colonization on the implanted catheter materials (Prateeksha et al., 2021). Under alkaline conditions, CP-AgNPs were synthesized by easily transferring electrons to form Ag^+^ ions due to the ionization of the ketone group of CP (Polte et al., 2012). The loading efficiency of CP on AgNPs was about 21% ± 1.1%, and it could be continuously released from CP-AgNPs. Flow cytometry analysis showed that compared to the control groups, the internalization rate of CP-AgNPs in *Pseudomonas aeruginosa* PAO1 and *Escherichia coli* (*E. coli*) was higher. For bacteria treated only with CP, there was no CP in both bacteria, further proving that CP-AgNPs would be a potential cargo delivery platform that could modulate bacterial QS and biofilm formation. The downregulation of QS-related genes indicated that CP molecules effectively delivered through CP-AgNPs could resist QS. Subsequently, a stable and durable CP-AgNPs coating was prepared on the surface of commercial silicone and polystyrene urinary catheters, with a retention time of more than 10 days to avoid protein and cell adsorption and prevent QS effect. The mechanical characteristics of the coated urinary catheters were not affected by the CP-AgNPs coating, and they could be freely imported into and removed from the bladder. Their clinical application would not be limited by the surface roughness of CP-AgNPs coatings, so bacterial adhesion might be accordingly restricted. Finally, the authors also evaluated the anti-adhesion effect of the nanolayer coated-urinary catheters through *in vitro* and *in vivo* tests, and achieved satisfactory results.

Another promising bioactive molecule, periostin (POSTN), is a stromal cell protein found in some tissues in embryonic development and is believed to be able to continuously regenerate and repair human tissues (Kormann et al., 2020; Wang et al., 2021b). It was reported that POSTN could not only bind to integrin receptors to promote cell spreading, proliferation, and tissue repair, but also induce macrophage proliferation and M2-subtype macrophage polarization to enhance tissue regeneration and wound healing (Wu et al., 2015; Allard et al., 2018; Liao et al., 2020; Nikoloudaki et al., 2020). Chen et al. delivered POSTN into the bladder through gelatin methacryloyl (GelMA) granular hydrogel to evaluate the potential mechanism of bladder urothelial regeneration in acute cystitis induced by cyclophosphamide (CYP) (Zhihong et al., 2022). The authors first detected upregulation of the POSTN gene and protein in CYP-treated mouse bladder, indicating that POSTN could regenerate bladder tissues. While the lack of POSTN deteriorated the structure and function of CYP-treated mouse bladder, delayed the regeneration of umbrella cells, and thus hindered the repair of the bladder barrier. The immunofluorescence staining of proliferation markers (pHH3 and Ki67), mRNA expression of differentiation markers for urothelial stem cells (Wnt1, Wnt2, Wnt2b, etc., and c-Myc, CCND1, AXIN2), as well as Western blot and immunofluorescence of urothelial stem cell marker (Krt14), showed that POSTN promoted the proliferation of urothelial cells by inducing AKT and Wnt4 and hence activated the signal of β-catenin. A large amount of CD68^+^, a lower level of CD86 (M1 marker) expression and a higher level of CD206 (M2 marker) in the bladder showed that POSTN promoted the proliferation of residual macrophages, and macrophages were polarized into the M2 subtype, providing a suitable microenvironment for regeneration after acute injury. After intravesical delivery of GelMA hydrogel loaded with POSTN into the CYP-treated bladders using a catheter, HE staining and immunostaining images demonstrated that the exfoliation of umbrella cells was reduced, and a large number of urothelial stem cells was observed, indicating that CYP treatment promoted the regeneration of urothelial and prevented bladder barrier injury. Therefore, this study concluded that POSTN could promote the proliferation of urothelial stem cells and macrophage polarization, and enhance the regeneration of CYP induced bladder injury. So it has a promising clinical application for patients with cystitis.

### 3.2 Growth factors

Angiogenesis involves the effective proliferation of endothelial cells and the formation of tubular structures (Herbert and Stainier, 2011). For example, VEGF can bind and activate the endothelial cell receptors, thereby enhancing cell migration and proliferation, and thus promoting the formation of blood vessels (Castro et al., 2018). Mokhames et al. (2021) fabricated electrospun polyvinylidene fluoride (PVDF) nanofibers, and further functionalized them with VEGF by neutralizing Col with sodium hydroxide. The prepared PVDF/Col/VEGF scaffold was fibrous and had interconnected pores with an average diameter of 900 ± 750 nm. After 168 h, the loaded VEGF continuously released up to 72%, and the PVDF/Col/VEGF scaffold exhibited significantly higher protein adsorption capacity than the PVDF/Col scaffold. The VEGF-loaded scaffold not only promoted the attachment and proliferation of primary bladder SMCs and HUVECs, but also significantly downregulated the apoptotic-related genes of SMCs (Bax, P53, P21, EP300, E2F5, and SMAD5) and upregulated angiogenesis-related genes of HUVECs (VEGFR1, VEGFR2, and CD31). The results indicated that the loading of VEGF in the PVDF/Col nanofibrous scaffold was helpful to manufacture tissue-engineered grafts for bladder wall reconstruction.

**FIGURE 5:**
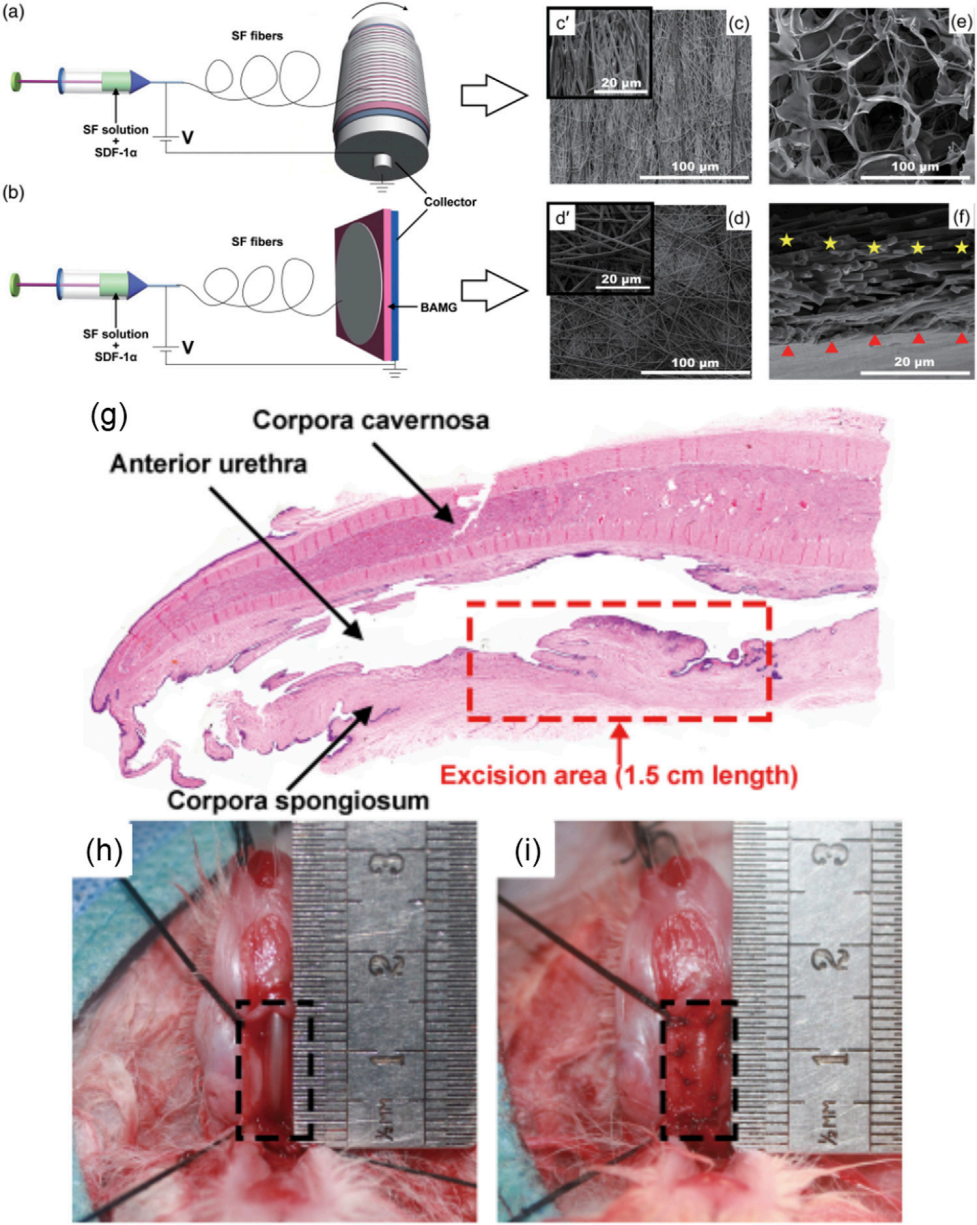
Schematic diagram showing electrospinning of the SF/3D-BAMG composite scaffolds with **(A)** aligned and **(B)** nonaligned fibers in aqueous solutions. Scanning electron microscopy images show the surfaces of [**(C)**, c’] aligned the SF microfiber and [**(D)**, d’] the nonaligned SF microfiber. **(E)** The surface of 3D-BAMG, and **(F)** cross section of the aligned SF/3D-BAMG composite scaffold. 3D-BAMG has been shown by a red triangle and silk microfiber has been indicated by yellow stars. **(G–I)** Substitution urethroplasty *in vivo* **(G)** Histological diagram of urethral defect rabbit model with an average length of a 1.5-cm ventral excision area. **(H)** Animal rabbit of the urethral defect. **(I)** Urethral reconstruction using composite scaffolds. Acta Biomaterialia. 2017; 61:101–113. Copyright 2020, with permission from Wiley (Liu et al., 2020b).

As an important chemotactic, stromal cell-derived factor-1 alpha (SDF-1α) was incorporated and sustained released from a composite scaffold to recruit stem cells for homing and trigger self-repair ability after urethral tissue injury (Tang et al., 2017; Liu et al., 2020b). Liu et al. fabricated the composite scaffold through electrospinning aligned or nonaligned SF microfibers containing SDF-1α on porous 3D BAM graft (3D-BAMG) (Figure 5) (Liu et al., 2020b). The aligned SF microfibers were arranged in the same direction, which was conducive to the continuity of cell extension and distribution directional. The nonaligned silk microfibers were arranged in multiple directions. Both aligned and nonaligned SF were porous structures with high porosity. The aligned or randomly arranged SF microfibers attached on the highly porous 3D-BAMG graft, which facilitated cell infiltration and growth, as well as the formation of blood vessels and smooth muscles. The support of 3D-BAMG effectively enhanced the mechanical property of the SF fibrous scaffold. After immersing in PBS for 16 days, the maximum release rate of SDF-1α was up to 8.5%, and the release amount was about 9.9 × 10^3^ pg. The controlled release of SDF-1α stimulated the migration of ADSCs and bone marrow stromal cells (BMSCs) cultured in a Transwell system for 16 days, showing the capacity to recruit and induce the chemotaxis of endogenous stem cells. Under urethroplasty, the aligned SF/3D-BAMG scaffolds functionalized by SDF-1α were implanted in a ventral urethral defect with a size of 1.5 × 1 cm^2^. Thicker epithelial layers and orderly arranged epithelial cells were formed in the group of SF/3D-BAMG scaffold containing SDF-1α. The number of smooth muscle fibers and vascularization in the submucosal area were also significantly promoted, delaying fibrosis as indicated by the decrease in collagen deposition in the SDF-1α-incorporated scaffold group. The findings from this study demonstrated that the successful loading of chemokine SDF-1α in the scaffolds efficiently regenerated urethral mucosa and activated the signaling pathway of SDF-1α/CXCR-4 to form new submucosal smooth muscles and microvessels (Yang et al., 2018; Sadri et al., 2022). It provided a promising candidate for reconstructing long urethral defects by accelerating *in situ* urethral regeneration without seeding any foreign cells. Furthermore, it was interesting to note that cytotoxicity was observed when the concentration of SDF-1α was up to 500 ng/mL. Therefore, it is necessary to further explore the effect of gradient concentrations of SDF-1α in the composite scaffolds on lone-term urethral regeneration and other related signaling pathways.

To overcome the fibrosis and graft contraction of traditional scaffolds, promote angiogenesis and the regeneration of smooth muscle, urothelial, and neuromuscular, and finally enhance bladder augmentation, a variety of growth factors, including basic fibroblast growth factor (bFGF), VEGF, and EGF, were utilized to functionalize gradient PCL scaffold (Freeman et al., 1997; Kanematsu et al., 2003; Nillesen et al., 2007; Kim et al., 2020b). In this study, Kim et al. (2020b) first produced gradient PCL scaffold by immersing them in a hot solution of 90°C PCL/tetraglycol in ethanol at 17 C for 1 h. The decrease in solubility led to the precipitation of PCL scaffolds, accompanied by the generation of gradient structure that became looser from bottom to top in a vertical direction, which was conducive to cell differentiation by providing a large surface area for cell growth (Di Luca et al., 2016). The loaded growth factors could be sustainedly released for 24 days without initial burst release from the complex path of the labyrinthine-like gradient structure, thus avoiding the chemical conjugation process using harmful reagents (Zhang et al., 2009; Gao W et al., 2021). After implanting into the bladder wall defect of partial cystectomy for 12 weeks, the growth factors-incorporated PCL scaffold almost restored the original volume of the bladder, with a maximum volume of 1,650 ± 300 μL, which was significantly larger than the volume of the PCL scaffold (1,291 ± 443 μL). Histological evaluation showed that compared to the scaffold without growth factors, the PCL scaffold containing growth factors regenerated well-organized urothelial layers, thicker and denser smooth muscle bundles, tighter connective tissue, and blood vessels. The results of immunohistochemistry staining and expression of genes related to the differentiation of smooth muscle and squamous urothelium further proved that the growth factors loaded in the PCL scaffold could induce the endogenous stem cells to differentiate towards urothelium and smooth muscle, which benefited from the paracrine effect of the functionalized scaffold. The bFGF and VEGF activities of the scaffold also greatly enhanced the regeneration of the neuromuscular junctions in contact with neurons, thereby maintaining homeostasis as evidenced by the high positive staining of α-bungarotoxin. In addition, anti-CD8 antibody staining revealed that the immune response of the growth factor-functionalized scaffold was lower than that of the PCL scaffold group, and the inflammatory level of the latter was similar to that of the partial cystectomy group. This study demonstrated that the continuous release of growth factors preserved the biological activity of the PCL scaffold and enhanced bladder regeneration and long-term *in vivo* safety.

Compared with the combination of growth factors and materials, Bates and his colleagues injected KGF directly into mice through subcutaneous injection to understand the regeneration mechanism of bladder injury caused by cyclophosphamide (Narla et al., 2020). That’s because the administration of cyclophosphamide has a potential risk of inducing bladder fibrosis or urothelial carcinoma (Vlaovic and Jewett, 1999). The authors found that KGF could inhibit the apoptosis of bladder cells by activating AKT signaling, thus preventing apoptosis of extravesical cells after cyclophosphamide administration (Fehrenbach et al., 2000). Besides, the injection of KGF also promoted the proliferation of KRT5^+^/KRT14^-^ urothelial cells on the lumen surface composed of newly formed superficial cells driven by ERK signaling, thereby enhancing the regeneration of epithelial cells (Ornitz and Itoh, 2015). Altogether, KGF reduced damage, led to early urothelial regeneration, and recovered from cyclophosphamide-induced bladder injury.

As an effective mitogen, bFGF contributes to angiogenesis, wound healing, and tissue regeneration (Nakamichi et al., 2016; Abdelhakim et al., 2020; He et al., 2022). To overcome the shortcomings of short half-life and fast diffusion of bFGF in medical applications and improve its binding efficiency with biomaterials, researchers engineered a bFGF fusion protein containing collagen-binding domain (CBD-bFGF) using gene recombination technology. And thus due to the introduction of CBD motifs, bFGF acquired significant binding capacity with collagen materials, while the biological activity of CBD-bFGF was not affected (Zhao et al., 2007; Wu et al., 2018). Dai and his colleagues first introduced von Willeband’s factor (vWF)-derived WREPSFCALS and collagenase-derived TKKTLRT amino acid residues into natural bFGF to recombine two fusion proteins, V-bFGF and C-bFGF, respectively, for targeted therapy of wound repair with less dosage of growth factors (Desouza and Brentani, 1992; Nishi et al., 1998; Andrades et al., 2001; Zhao et al., 2007). Both V-bFGF and C-bFGF maintained similar biological activity and strong collagen binding activity similar to natural bFGF due to no intramolecular disulfide bonds were formed in the loosen 3D secondary structure composed of 11 β-sheets avoiding the interface between bFGF and CBD motifs (Eriksson et al., 1991). The collagen-binding ability of the fused C-bFGF was higher than that of V-bFGF, and it had better cell integration and angiogenesis, which was conducive to targeted cellular function and tissue regeneration.

Subsequently, Dai’s team successively used the CBD-bFGF-modified collagen scaffolds to regenerate injury of bladder, uterine horns, abdominal wall, extrahepatic bile duct, sciatic nerve, and tympanic membrane perforation (Chen et al., 2010; Li et al., 2011; Shi et al., 2011; Li et al., 2012; Ma et al., 2014; Zhang et al., 2017). Among them, to establish a targeted delivery system of bFGF protein in clinical application, Chen et al. (2010) transplanted CBD-bFGF functionalized collagen scaffolds onto the residual bladders after partial cystectomy in rats. Histological examination showed that the regenerated bladder had good structure, newly formed blood vessels and ingrowth of SMCs. The recovery of bladder volume capacity and compliance as evidenced by urodynamics implied satisfied bladder tissue regeneration by the functional collagen materials. Further, to provide sufficient evidence for the regenerative ability of human bladder injuries, the same authors conducted *in vivo* repair of damaged bladder in large animals. In this work, Dai and his colleagues first prepared BAM scaffolds through decellularization, and then immobilized the fused CBD-bFGF containing a collagen-binding peptide of TKKTLRT onto the scaffolds (Shi et al., 2017). *In vitro* evaluations showed that CBD-bFGF efficiently bind to natural BAM in a dose-dependent manner, and could be sustainedly released for 14 days without burst release. The functional CBD-bFGF/BAM scaffolds were sutured to the rest half bladder of beagles that underwent hemicystectomy. The urodynamics test showed an improvement in bladder contractility, with a maximum bladder volume of 41.50 ± 5.35 for the CBD-bFGF/BAM regenerated bladder. The PBS/BAM group showed a statistical lower volume (28.28 ± 4.64, *p* < 0.01). Similar bladder compliance was observed in the CBD-bFGF/BAM group (0.94 ± 0.13), which was superior to the PBS/BAM group (0.55 ± 0.10, *p* < 0.01). The CBD-bFGF/BAM group obtained a smooth bladder similar to the sham operation group, but different from the PBS/BAM group, there were some rough texture areas. The CBD-bFGF/BAM group integrated with adjacent tissue, with a dense urinary epithelial cell layer, and formed well-arranged smooth muscle bundles on the bladder wall as in the sham operation group. It was worth noting that compared to the PBS/BAM group, the CBD-bFGF/BAM group showed a significant increase in neovascularization and regenerated nerve fibers, and no inflammatory reaction was detected. In summary, due to the targeted delivery and controlled release of bFGF at the target site, the functionalized CBD-bFGF/BAM scaffolds could more efficiently utilize bFGF to reconstruct bladder tissue and reduce side effects in the dog models.

Nevertheless, the exact mechanism of bladder regeneration using CBD-bFGF functionalized scaffolds is still unclear, and further research is needed on bladder repair to evaluate the histological and functional recovery of smooth muscle, nerves, blood vessels, and urothelium. More importantly, multiple growth factors and stem cells can be combined to better regenerate bladder tissue (Kim et al., 2021; Horii et al., 2022).

Scaffolds for efficient delivery and controlled release of TGF-β have also been developed to regulate stem cell differentiation and promote the regeneration of target injured tissues (Chen et al., 2020; Ye et al., 2022). For instance, Ardeshirylajimi et al. prepared electrospun nanofiber scaffolds composed of PVDF and chitosan nanoparticles loading with TGF-β for bladder regeneration (Ardeshirylajimi et al., 2018). Under SEM it was observed that the average diameter of the electrospun smooth nanofibers was 845 ± 456 nm. The plasma treatment increased the hydrophilicity of the nanofibrous scaffold, reducing the contact angle to 42°, much lower than 138° of untreated nanofibers. The MTT results showed that the proliferation of ADSCs cultured on the PVDF scaffold was significant better than that of tissue culture polystyrene (TCPS). The release kinetics showed that 70% of the loaded TGF-β continuously released from the nanofibers within 2 weeks, it significantly promoted the viability and proliferation of ADSCs. The anti-inflammatory function was observed for both the PVDF and PVDF/TGF-β nanofibers when HUVECs were monolayer cultured on the scaffolds using transwells and treated with lipopolysaccharide (LPS). The upregulated gene expression of SM-22α, calponin-1, α-SMA, and protein expression of α-SMA indicated that the TGF-β-functionalized PVDF nanofibrous scaffold had superior SMC differentiation potential in clinical bladder regeneration.

Platelet-rich plasma (PRP) is widely noticed by researchers and clinicians because it is rich in a variety of growth factors and cytokines, such as insulin-like growth factor 1 (IGF-1), bFGF, EGF, VEGF, TGF-β, and PDGF (Marques et al., 2015; Ziegler et al., 2019; Poulios et al., 2021; Khodamoradi et al., 2022). It has been reported that the injection of PRP could promote the proliferation of urothelial cells and the expression of cytoskeleton and urinary barrier function protein in the treatment of recurrent urinary tract infection (Jiang et al., 2021). PRP could also induce angiogenesis, promote the regeneration of bladder mucosal and nerves, improve the recovery of erectile function, and avoid SMC apoptosis (Mirzaei et al., 2019b). In one study, Juan and his colleagues prepared rat PRP and perfused it into the bladder of ketamine induced ulcerative cystitis once a week for 4 weeks (Chueh et al., 2022). Compared with the ketamine treatment group, the PRP treatment group significantly reduced the frequency of urination, decreased the peak pressure of urination, and expanded the bladder capacity, urine volume, and urination interval. In PRP-treated bladder tissues, the immunostaining distribution of the proliferation marker Ki67 in the basal layer of urinary tract epithelium demonstrated that PRP had a mitotic effect of stimulating mucosal proliferation and improving mucosal regeneration. What’s more, the protein expression level of proliferation markers and urothelial tight junction markers was upregulated by the PRP treatment. All these findings showed that PRP treatment could promote bladder regeneration by stimulating cell proliferation and differentiation. The expression of α-SMA, one of the angiogenesis-related proteins, was increased by the injection of PRP, and it could stimulate the angiogenesis of the bladder. PRP treatment also enhanced the regeneration of intramural nerve and alleviated the oxidative damage in ketamine-induced bladder injury. The perfusion of PRP *in vivo* verified the biosynthesis of inflammatory fibers, the transformation of fibroblasts into myofibroblasts, anti-inflammation, the promotion of cell proliferation, angiogenesis, and neurogenesis to restore bladder function and repair bladder. This preclinical trial of PRP in the treatment of bladder injury not only elucidated the potential pathophysiological therapeutic mechanisms of PRP on bladder dysfunction and tissue remodeling, but also provided reference for the clinical application of PRP, such as the concentration of growth factors and platelets, standardization and quality management of PRP formulations (Fadadu et al., 2019; Andia et al., 2020).

### 3.3 Antibody

The low survival rate of cells directly seeded on the implanted patch for urological repair has aroused increasing concern about the safety and even effectiveness of such cell-loading therapy (Amer et al., 2017; Pokrywczynska et al., 2019). Song et al. designed functional scaffolds by conjugating anti-CD29 antibody onto SIS patch (AC-SIS) to selectively capture autologous urine stem cells (USCs) *in situ* for bladder regeneration (Song et al., 2022). Specifically, two-step reaction effectively enabled the chemical binding of the anti-CD29 antibody and SIS patches. The grafting rate of anti-CD29 antibody on the AC-SIS scaffold was as high as 72%, and it did not affect the biocompatibility of the scaffold. After 1, 3, and 5 days of culture, the proliferation of USCs on the AC-SIS was superior to that of the pure SIS group. under dynamic conditions, the AC-SIS group captured more cells than the SIS group. After 8 weeks of implantation of the scaffolds into full-thickness bladder defect, the bladder wall of AC-SIS transplantation showed a thickness similar to that of a normal bladder wall. While the bladder wall in the control group was thinner, slightly larger in size, and less elastic than normal tissue. The bladder size in the AC-SIS group was about 5 cm × 5.3 cm, closest to a normal bladder. The histological analysis revealed that the implanted AC-SIS samples facilitated the regeneration of urothelium, forming a unique hierarchical structure similar to the natural bladder urothelium composed of loop fluctuations and smooth muscle fibers. The captured USCs captured by the AC-SIS scaffolds altered the microenvironment, leading to vascular growth, re-epithelization, collagen and smooth muscle bundle formation. All of these aspects presented important potential for structural reconstruction, bladder internal pressure maintenance, and bladder function integrity.

### 3.4 Nucleic acid

It has been noted that the number of stem cells that differentiated into SMCs was very small and most of the stem cells inoculated on the scaffolds remained undifferentiated (Pokrywczynska et al., 2018). To increase the myogenic differentiation of stem cells and help reconstruct bladder function, Jin et al. designed lipid nanoparticles containing RNA activation targeting the MyoD promoter (saRNA), and construct composite scaffolds [NP(saMyoD/BAMG)] to promote the upregulation of endogenous gene expression (Jin et al., 2020). In this study, the ADSCs transfected with NP(saMyoD) upregulated the expression of Desmin, SM-22α, and α-SMA. The efficient delivery and releasing of the saRNA through NPs regenerated bladder smooth muscle and enhanced neovascularization, as demonstrated by the well-regenerated urothelium, dense arrangement of smooth muscle fibers, and the formation of blood vessels, thereby improving bladder defect repair and urinary function. It provides a new perspective for developing alternatives for bladder defect repair.

**FIGURE 6:**
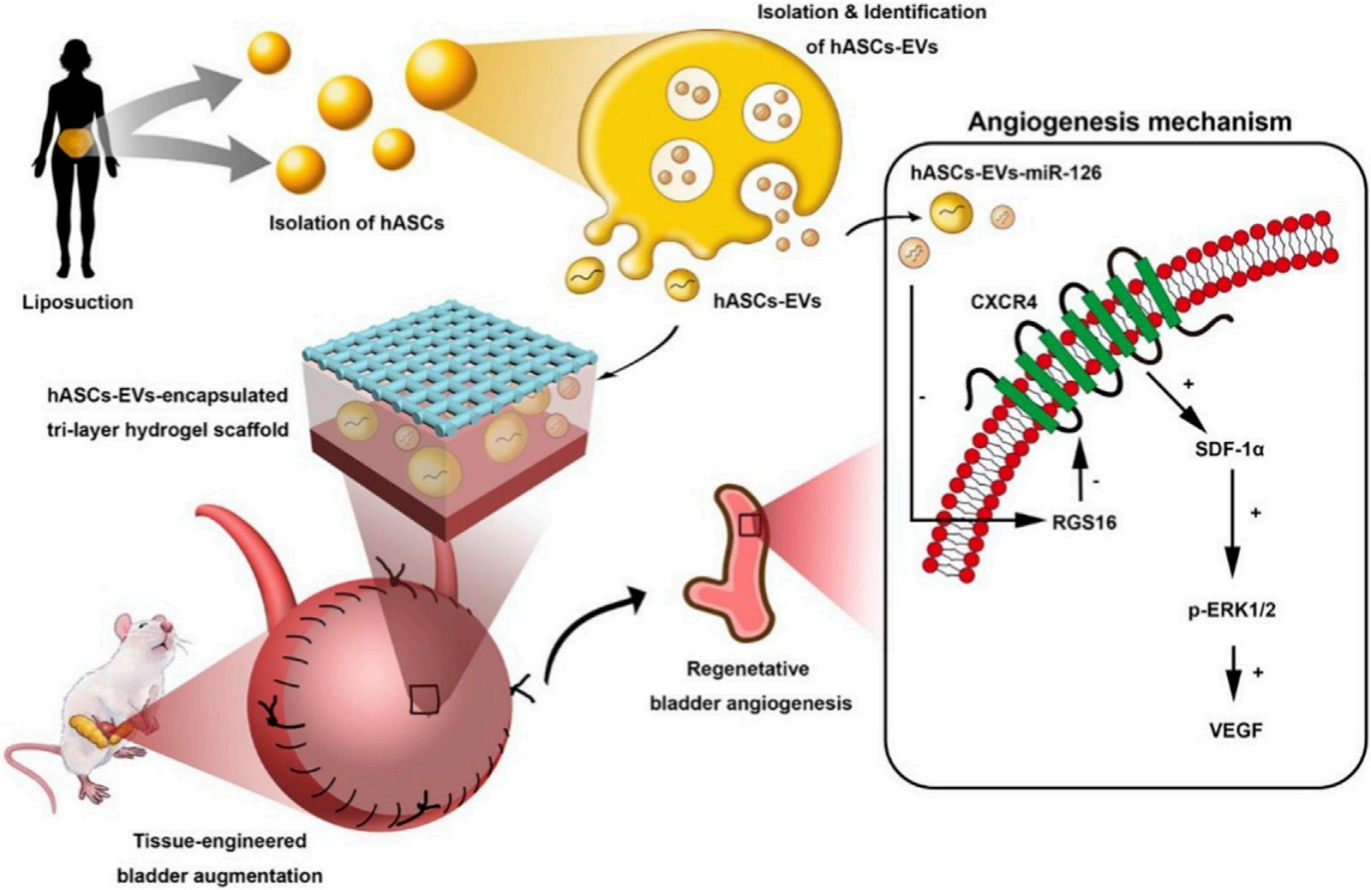
Schematic illustration of Human ASCs-EVs-encapsulated BAMG hydrogel scaffold delivers miR-126 to promote bladder regeneration angiogenesis through CXCR4/SDF-1α activation. *Chemical Engineering Journal.* 2021; 425:131624. Copyright 2021, with permission from Elsevier (Xiao et al., 2021a).

In another work, microRNA-126 contained in the extracellular vesicles (EVs) derived from human ADSCs was incorporated into BAMG-based hydrogel scaffolds by Xiao et al. (2021a) to facilitate bladder regeneration (Figure 6). The bladder defect was reinforced by the sufficient mechanical characteristics of the degradable scaffold and accordingly regenerated by the effective delivery of the human EVs to the injury site. The internalization of miRNA-126 in HUVECs promoted angiogenesis by inhibiting G-protein signaling 16, and activated the CXCR4/SDF-1αpathway, thereby secreting VEGF through the phosphorylation of ERK1/2. Finally, the formation of well-organized urothelium, smooth muscle, tube-like structure, and neural fibers for bladder augmentation, improved the morphology of regeneration and functional recovery.

### 3.5 Physical factors

It was reported that low-intensity extracorporeal shock wave therapy (LiESWT) could promote penis tissue regeneration and regulate penis hemodynamics in patients with erectile dysfunction (ED) (Chung and Wang, 2017). Lin et al. evaluated the therapeutic effect of LiESWT on bladder tissue vascularization, inflammatory response regulation, and improvement of bladder hyperactivity in ovarian hormone deficiency (OHD)-induced overactive bladder (OAB) in the human body and rat model (Lin et al., 2021). After 8 weeks of treatment with LiESWT (0.25 mJ/mm^2^ and 3,000 pulses), the authors found that an increase in regeneration of bladder urothelium, inhibition of interstitial fibrosis, promotion of cell proliferation, increased expression of angiogenesis-related proteins, and increased protein phosphorylation levels of Akt, P38, and ErK1/2. Accordingly, the frequency of urination decreased, residual urine volume after urination and urgent incontinence also reduced, while the maximal flow rate and urine volume increased. In this study, the potential molecular mechanism of applying LiESWT might be regulating bladder overactivity, reducing inflammatory reaction, increasing neovascularization, enhancing cell proliferation and differentiation, and thereby improving the life quality of postmenopausal patients.

## 4 Innovative approaches

### 4.1 3D printing

**FIGURE 7:**
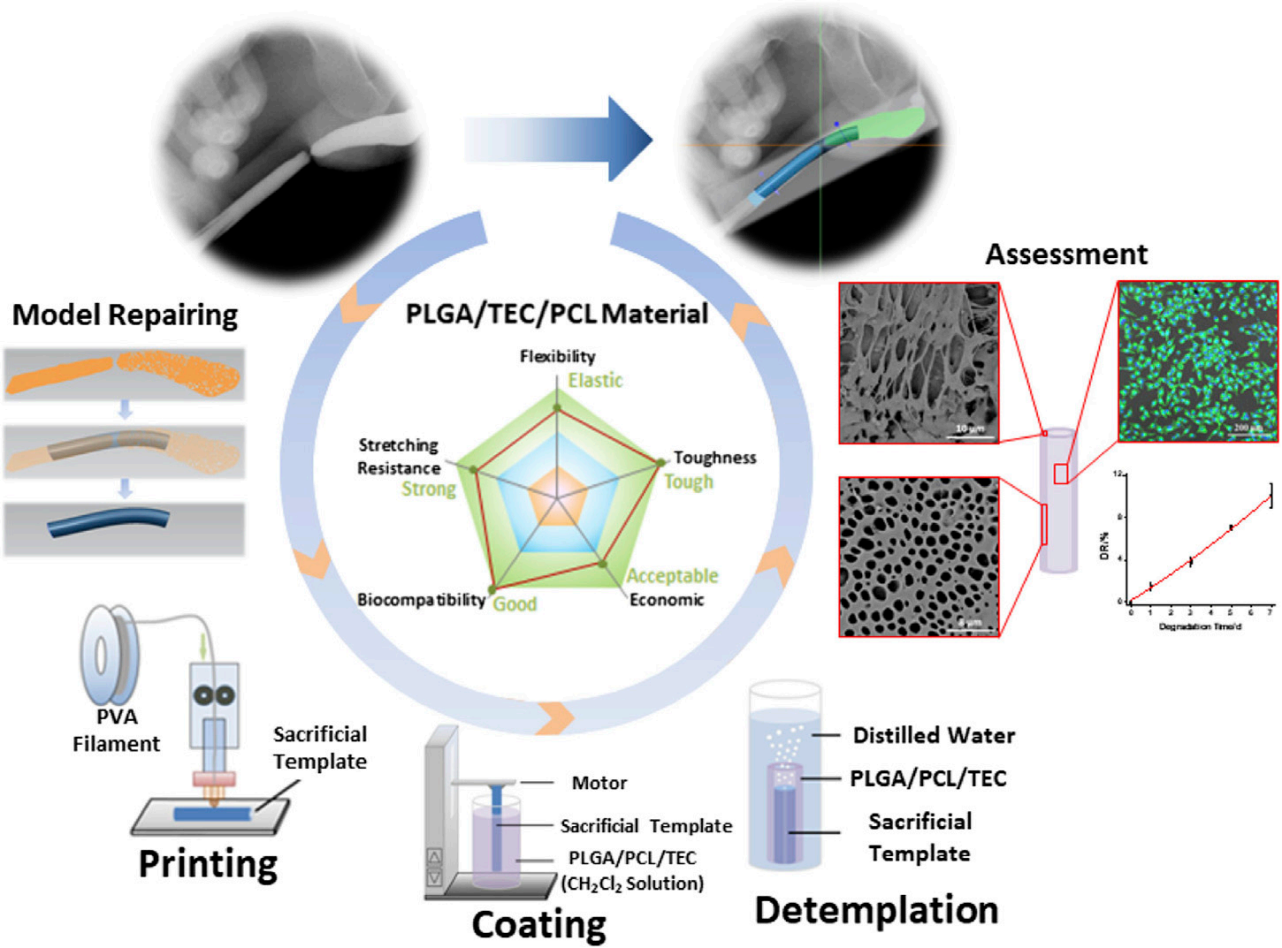
Manufacturing process of a biodegradable scaffold for urethra tissue engineering based on 3D printing. *ACS Applied Bio Materials.* 2020; 3: 2007–2016. Copyright 2020, with permission from American Chemical Society (Xu et al., 2020b).

The diagnostic and therapeutic strategies for bladder dysfunction and tissue damage are often hampered by the lack of reliable *in vitro* 3D models to simulate the complex features of the human bladder. To overcome this problem and improve precision treatment, Xu et al. (2020b) prepared porous PLGA/PCL composite scaffolds incorporating different contents of triethyl citrate (TEC) using 3D printed polyvinyl alcohol (PVA) sacrificed templates for urethra tissue engineering (Figure 7). The mechanical characteristics of the PLGA/PCL scaffolds were influenced by different levels of PCL. Due to the inherent toughness of PCL and the poor phase interface between the two incompatible phases, the tensile strength of PLGA/PCL with higher PCL content (0%, 10%, 20%, 30%, 40%, and 50%) gradually reduced from 12.60 to 8.53 MPa. Meanwhile, due to the toughness of PCL, the Young’s modulus of PLGA/PCL gradually increased from 121.08 to 178.88 MPa, making the materials more difficult to deform. Adding TEC into the PLGA/PCL (70:30) composite could effectively promote the compatibility between PLGA and PCL phases, with a maximum tensile strength of 11.11 MPa at 6% TEC. L929 cells with growth behavior similar to the urothelial cells were cultured on the PLGA/PCL/TEC (70:30:6) samples, and their fluorescence staining morphology and proliferation showed good biocompatibility and low cytotoxicity. The PLGA/PCL/TEC (70:30:6) materials also exhibited a moderate degradation rate between PLGA and PCL, indicating its suitability and feasibility as scaffolds for urethral tissue engineering.

In addition, 3D bioprinting and stereolithography (SLA) 3D printing techniques have also been utilized to create *in vitro* 3D urinary bladder models. In one study, Chae et al. reconstructed the physiological microenvironment of the bladder using a 3D bioprinting platform combined with decellularized bladder ECM and periodic mechanical stimulation (Chae et al., 2022). The created bladder models showed high cell viability and proliferation efficiency and enhanced the promotion of myogenic differentiation under dynamic mechanical stimulation. It envisions proposing a meaningful *in vitro* bladder model as a platform for drug screening, disease diagnosis and treatment. In another study, the authors used SLA 3D printing to prepare an elastic polymer-based indwelling bladder devices for the first time to achieve controlled local delivery of lidocaine hydrochloride (Xu et al., 2021). With the help of a urethral catheter, the hollow and solid bladder devices inserted into and retrieved from the bladder showed good blood compatibility, and the resistance to compression and tension was satisfactory, and they returned to their original shape immediately after removing the external force. Lidocaine was sustained released from the solid devices for 14 days. That is to say, as a revolutionary strategy, SLA 3D printing technology can manufacture drug delivery devices for the treatment and regeneration of bladder disease.

For urinary tissue engineering, 3D bioprinting using hydrogels containing cells as bioink is more suitable for manufacturing personalized biomimetic tissues than ink based only on biomaterials (Xu et al., 2022). Accordingly, 4D bioprinting invloving hydrogels based on intelligent stimulus response polymers as bioinks have been developed to manufacture urinary implants, as well as on demand stimulus responsive drug delivery. Various hydrogel materials with appropriate 4D bioprinting performance also have great potential in urinary tissue engineering, including poly(N-isopropylacrylamide) and poly(N,N-dimethylacrylamide), alginate, etc. (Dong et al., 2020; Imam et al., 2021).

### 4.2 Organoids

The characteristics of organoids simulated organs *in vitro* provides a novel approach for drug screening and precision medicine, and have become a research hotspot (Vasyutin et al., 2019; Jeong et al., 2023). Organoids derived from pluripotent stem cells, adult stem cells or tumor cells can be reconstructed to recapitulate the critical characteristics of organs and tissues. Kim et al. (2020a) created bladder assembloids to mimic bladder tissue regeneration and cancer microenvironments. In this study, multi-layered bladder “assembloids” were constructed using stromal stem cells, and well-organized architecture was represented with stroma surrounded by muscle and epithelium layer. The assembloids exhibited the characteristics of cellular compositional and single-cell transcriptome gene expression level of human mature bladder, and recapitulated tissue dynamics *in vivo* in response to tissue damage.

Human urothelial organoid models are of great significance for the long-term tolerance of human cells to urine, especially for the interaction of pathogens in urinary tract infections. Therefore, Horsley et al. developed a novel human organoid from progenitor cells, which reconstituted the important structure and biomarkers of urinary tract epithelium (Horsley et al., 2018). After 3 weeks of transwell culture with urine, multilayer organoids with umbrella-like cell connections, asymmetric membranes, as well as glycosaminoglycan layers were reconstructed. *Enterococcus faecalis* infection showed invasive results similar to those of patient cells, including urinary tract epithelial exfoliation and intracellular colony formation. Therefore, with the help of this bionic organoid model, which is helpful for diseases diagnosis and treatment, it is possible to elucidate the invasive behavior of urinary tract pathogens. Further, more progress in 3D tissue culture will increase the fundamental research and development of human organoid models related to bladder physiology.

Ureteral stricture is a common and frequently-occurring disease in adults and children, requiring ureteral reconstruction. Takagi et al. fabricated a novel type of artificial ureter grafts by combining spheroids and 3D bioprinting techniques (Takagi et al., 2022). The spheroids were generated by co-culturing human dermal fibroblasts and HUVECs, and were later laminated using a 3D bioprinter. Then, after the laminated spheroids matured, a tubular structure was formed, which was then transplanted into the rats as an artificial ureter. At 12 weeks after surgery, the ureteral epithelium and muscle layer regenerated well. This means that the ureteral structure based on the cell-only stacked spheroids is conducive to the regeneration of short ureters and lays the foundation for complete ureterregeneration (Ryosaka et al., 2022).

The design and manufacture of organoids based on cell extrusion bioprinting can describe specific events during the invasion of urinary diseases, and can also improve the reproducibility of kidney organoid (Lawlor et al., 2021; Vandana et al., 2023). During this process, an organized urothelium model can completely regenerate with mature urothelial layer *in vitro*. By controlling the quality of kidney-like organ production, kidney tissue sheets with uniform patterns and tubular segments can also be produced (Jiang et al., 2023). Organoids also enables us to carry out further complex research by successfully simulating the tissue architecture and cellular disposition of normal bladder *in vivo*. In addition, organ-on-a-chip platforms combining organoids and microfluidic systems will help to establish more complex experimental models of multiple organ systems, and conduct efficient and economical toxicity tests, drug discovery, and drug screening (Berlo et al., 2021; Homan, 2023; Zhu et al., 2023).

## 5 Future prospects

Urinary system reconstruction is a medical mission with great challenges and far-reaching significance, because there are a large number of patients with complex tissue damage and dysfunction caused by congenital or acquired reasons in clinical need of repair. However, tissue engineering strategies involving functional biomaterials and biotechnology offer potential prospects for reconstructing damaged organs and tissues. Urologists urgently need to seek more technical and commercial product support from fields such as materials science, life sciences, biotechnology, and engineering. On the basis of vigorously promotig scientific and technological innovation in China, various tissue repair material products and biotechnology will increasingly benefit patients. One of the commercial products is a composite material composed of fibrinogen and poly(lactide-co-caprolactone) (PLCL) developed by Shanghai PINE&POWER Biotech Co., Ltd. This composite material was approved by the China Food and Drug Administration (CFDA) in August 2018 (Registration Certificate No. 20183130292), which is a tissue engineering electrospun scaffold similar to natural extracellular matrix. It has been successfully verified its good effect in bladder and ureter reconstruction and open, tension-free inguinal hernia repair (Li et al., 2019). Allium ureteral stent, developed by Allium Medical Solutions Ltd. (Allium^®^), is a self-expanding large caliber stent, made of a hyperelastic alloy covered by biocompatible polymer for preventing tissue ingrowth. The metal component provides radial and longitudinal strength, while the polymer bio-inertness prevents tissue from growing inward into the lumen and early encrustation (Gao CZ et al., 2021).

Significant efforts are still needed to achieve more successful commercial products for functional biomaterials and biotechnology. More attention should be paid to the biomimetic structure, appropriate mechanical properties, and biological functions of implants. What’s more, in order to achieve satisfactory repair quality, it is also necessary to address the issues of *in situ* vascularization and integration between the implants and the host tissue. For these goals, it is required to continuously conduct high-quality clinical trials in accordance with laws and regulations. Although there is still a long way to go, people believe that the clinical transformation of functional biomaterials and biotechnology will ultimately overcome the risks and promote the structural and functional outcomes of the urinary bladder, urethra, and ureter.

## 6 Conclusion

In this review, we aim to summarize the latest advances of bioactive materials and biomimetic technologies in urinary tissue engineering. A series of biomaterials, including decellularized tissues, natural and synthetic biopolymers, as well as hybrid scaffolds, were discussed. Subsequently, the biological functions of biomaterials utilizing growth factors, drugs, inorganic nanomaterials, etc., were reviewed. In addition, the biomimetic approaches of 3D (bio)printing and organoids in urinary system structure and function regeneration were also discussed. This review will present new ideas and insights for the innovative development of bioactive materials and biomimetic technologies in the field of urological tissue engineering.
